# Lower baseline serum neuronal-specific enolase levels predict better rate of recovery of functional walking ability in post-acute stroke patients

**DOI:** 10.1007/s10072-025-08723-5

**Published:** 2025-12-25

**Authors:** Stefano Brunelli, Sami Nourbakhsh, Lorenzo Grimaldi, Alessandra Calvani, Noemi Gentileschi, Roberta Mucci, Eleonora Bovi, Emilia Giannella, Sofia Toniolo, Gisella Guerrera, Luca Battistini, Domenico De Angelis, Giulia Sancesario

**Affiliations:** 1 NeuroRehabilitation Unit 4, IRCCS Santa Lucia Foundation, Via Ardeatina, 306/354, Rome, 00179 Italy; 2 Physical and Rehabilitation Medicine, Tor Vergata University, Rome, Italy; 3 Biobank, IRCCS Santa Lucia Foundation, Via Ardeatina, 306/354, Rome, 00179 Italy; 4 Experimental Neuroscience, European Center for Brain Research, Via del Fosso di Fiorano, 64, Rome, 00143 Italy; 5 Nuffield Department of Clinical Neurosciences, University of Oxford, Headington, Oxford, OX3 9DU UK; 6 Cognitive Disorder Clinic, John Radcliffe Hospital, Headley Way, Headington, OX3 9DU, Oxford UK; 7 Neuroimmunology Unit, IRCCS Santa Lucia Foundation, Via Ardeatina, 306/354, Rome, 00179 Italy

**Keywords:** Stroke, Blood biomarkers, Neuronal specific enolase, Brain-derived neurotrophic factor, Rehabilitation, Neurodegeneration

## Abstract

**Introduction:**

Evaluate the association between functional recovery and a panel of specific neuronal biomarkers, in a cohort of stroke patients.

**Material and methods:**

Serum levels of neuronal specific enolase (NSE), neurofilament light chain (NfL), brain derived neurotrophic factor (BDNF), amyloid-β_42_ and β_40_ peptides (Aβ_42_/Aβ_40_ ratio) and total tau (t-tau), were measured in 20 patients within one month after stroke event (baseline, T0). After six weeks of extensive multimodal cognitive and motor rehabilitation (T1), levels of each biomarker were correlated with changes in clinical scales for disability and mobility (Barthel Index (BI), Rivermead Mobility Index (RMI), Functional Ambulation Categories (FAC), Fugl Mayer Assessment Upper Extremity (FMA).

**Results:**

Linear regression was performed to predict changes in clinical scales during follow up, according to baseline biomarkers levels. NSE at T0 was a significant predictor of improvement in FAC and RMI, where the higher the NSE concentration, the smaller the improvement. Therefore, baseline NSE explained 39% of the variation in FAC and 31% in RMI over time. No significant differences were observed with respect to other scales or other biomarkers.

**Conclusions:**

This exploratory study suggested that serum NSE may be a predictor of functional mobility recovery in post-acute stroke patients and represents a useful tool for patients’ stratification.

## Introduction

Stroke is the second leading cause of death worldwide, with high social and economical costs related to post-stroke motor, sensory and cognitive deficits [1]. Several factors affect stroke recovery, such as severity, lesion location, comorbidities, and variability given by the individual characteristics of the patient [2]. To improve the efficacy of care and rehabilitation plans, it is crucial to understand the biological mechanisms underlying stroke and identify reliable biomarkers that can predict recovery outcomes.

To date, a number of clinical scales and instrumental tests have been introduced in clinical settings to evaluate the recovery of specific functions (motor, cognitive, speech, etc.). In particular, the National Institute of Health Stroke Scale (NIHSS) [3] is a strong predictor of patient recovery after stroke and may predict the functional outcomes in terms of physical performance, activities of daily living, and Instrumental Activities of Daily Living (IADL) independence [4, 5]. Moreover, the predictive value of ischemic lesion volume and the brain area involved was widely investigated [6, 7]. In 2017, the Stroke Recovery and Rehabilitation Roundtable recommendations provided a consensus statement on biomarkers that are considered ready for inclusion in clinical trials, and others showing promising results, for monitoring post-stroke recovery, with attention to brain imaging, electroencephalography and stimulation techniques, but excluding blood and genetic markers [8]. Relatively few studies have evaluated the intrinsic physiological mechanism of neural repair and protection of brain tissues occurring in patients after stroke, and corresponding changes in blood and cerebrospinal fluid components [9, 10]. Blood is advantageous as a source of biomarkers due to its easy accessibility, non-invasiveness, and the ability to detect systemic changes associated with pathology [11, 12]. In stroke, several promising blood biomarkers, such as brain derived proteins like brain-derived tau (BD-tau), glial fibrillar acid protein (GFAP) [13], neurofilament light chain (NfL) and Neuron-specific enolase (NSE), as well as neurotrophic factors like brain-derived neurotrophic factor (BDNF) or inflammatory markers, have been associated with stroke recovery [14–16]. While these biomarkers have shown promising results as prognostic tools, their reliability is limited due to patient variability resulting from the type of stroke, demographics, co-morbidities and so forth [17].

NSE is a cytoplasmic enzyme involved in glycolytic energy metabolism in the brain, and its increase during injury is widely considered a nonspecific marker of neuronal damage. Several clinical studies and systematic reviews have investigated the predictive value of NSE on outcome in patients in the early phase after stroke, showing a positive correlation with severity at admission at emergency department, degree of disability and neurological worsening [18].

BDNF is a neurotrophic factor involved in repairing brain damage and maintaining synaptic plasticity [19]; after stroke, low BDNF levels are associated with poor long-term functional outcomes [20] and depression [21], whereas rehabilitation significantly correlates with BDNF increase [10]. Amyloid β_42_ and β_40_ peptides (Aβ_42_ and Aβ_40_), are well known as key mediators of brain damage involved in the pathogenesis of Alzheimer Disease (AD) other than a risk factor for aging-related vascular pathologies. Blood Aβ_42_ correlates with stroke progression and post stroke cognitive impairment [22], whereas Aβ_40_ levels are elevated in stroke patients as consequence of ischemic insults [23].

Tau and NfL are neuronal proteins and circulating marker for neuroaxonal damage, often assessed to monitor neurodegenerative and neuroinflammatory conditions like AD and Multiple Sclerosis. Notably, increased tau concentrations have been shown to be significantly associated with unfavorable outcome after wide brain injuries, both traumatic as ischemic stroke [24, 25], suggesting a promising clinical use as blood-based predictive prognostic biomarker.

However, in cerebrovascular conditions, NfL has potential as a biomarker for determining infarct size in ischemic stroke, but not significant association as prognostic marker [26–28]. Studies have shown that acute and 3-month serum NfL concentrations correlate with infarct volume and time since stroke, with levels peak around 1 month, decreasing by 50% at 3 months and 99% at 9 months [29].

However, the potential of these markers to monitor or predict outcomes and recovery during extensive rehabilitation in the post-acute phase of stroke, when patients are clinically stable, has never been investigated.

In this exploratory study, we evaluated the association between functional recovery and a panel of serum proteins, including NSE, BDNF, Aβ_42_, Aβ_40_, tau and NfL, aimed at exploring their potential value as predictive and prognostic biomarkers in a cohort of post stroke patients.

## Patients and methods

### Subjects

The study took place at Neuro-Rehabilitation Unit 4 of Fondazione Santa Lucia, Rome, Scientific Institute for Hospitalization and Care (IRCCS) specialized in neurorehabilitation. We screened all inpatients with recent ischemic stroke that were consecutively admitted to our facilities for a period of 18 months, from January 2023 to June 2024. The patients were transferred from the Stroke Units of the local Hospitals once their clinical conditions had stabilized, generally after 7 days and within a maximum of 30 days from the initial event. Subjects were recruited according to the following inclusion criteria: patients experiencing their first episode of ischemic stroke, adult age, enrolment within a maximum of 1 month after the acute event, no other disabilities measured with modified Ranking Scale (mRS = 0) administered anamnestically [30]. Patients with hemorrhagic stroke, severe cognitive or psychiatric deficit or unstable clinical conditions, with previous diagnosis of dementia, or other neurological conditions, were excluded from the study. All patients underwent neuromotor reeducation treatment, consisting of two treatments per day of 40 min each with a frequency of five times a week. The neuromotor re-education treatment consisted of the following activities: facilitation of movements on the paretic side, muscle tone and muscle compensations control, strengthening exercises, stretching exercises, trunk stabilization, balance training, standing, sitting and transferring task, conventional assisted overground walking and occupational therapy aimed at recovering autonomy in the activities of daily life. If necessary, the patient was also treated with speech and attention deficit therapy and swallowing deficit training.

### Clinical functional scales

The following scales were assessed: Functional Ambulation Classification (FAC) for walking ability; the Rivermead Mobility Index (RMI) for functional mobility; the Barthel Index (BI) for the disability in activities of daily living by; the Fugl-Meyer Assessment Upper Extremity (FMA) for the severity of impairments in motor function of the upper paretic limb. FAC is a 6-point ordinal scale used to evaluate a patient’s walking ability based on the level of physical support required. Scores range from 0 to 5, where 0 indicates the patient is non-ambulatory or requires assistance from two or more persons, and 5 indicates the ability to walk independently in all environments, including stairs and uneven terrain. Intermediate scores represent varying degrees of dependency during ambulation. In detail: FAC 0- the patient cannot walk or requires help of 2 or more people; FAC 1- the patient requires firm continuous support from 1 person; FAC 2- the patient needs intermittent or continuous light touch to assist balance or coordination; FAC 3- the patient is able to walk without physical contact; FAC 4- the patient can walk independently on level ground, but requires help on stairs, inclined or uneven surfaces; and FAC 5- the patient can walk independently [31].

RMI investigates the functional mobility in gait, balance and transfers and includes 15 mobility items with dichotomous answers (yes = 1/no = 0). Thus, the cumulative score may range from 0 to 15, with a higher score indicating better patient mobility [32]. The RMI score reflects the patients’ ability to move their own body within the domestic environment. It comprises evaluations of basic abilities like “turning over in bed” or “lying to sitting” but also more complex abilities like “walking up and down four steps” or running.

The BI is a 10-item ordinal scale that covers mobility (like transfers bed to chair and back or mobility on level surfaces) and self-care domains (like feeding, bathing, dressing); scores range from 0 (total dependence in ADL) to 100 (complete independence). We used the modified version of Shah et al. 1989 [33].

FMA is a widely used assessment tool in neurology for quantitative measurements of post-stroke recovery of upper extremity in hemiparetic/hemiplegic patients [34]. It comprises a motor score section (FMA-MS) that includes volitional movement of the shoulder, elbow, wrist and hand as well as coordination tasks. The FMA-MS score ranges from 0 (no muscle activity) to 66 (normal activity). The full FMA also covers the assessment of pain, sensations, and joint range of motion. Final scores of full FMA range from 0 to 126. Blood samples were collected and clinical scales were assessed at enrollment (T0, baseline), and after 6 weeks of intensive rehabilitation (T1). Assessors were blinded to the biomarker levels during clinical evaluation.

### Neurological biobank

Specific informed written consent for participating in the study was obtained for all patients. Further, consent was requested for donation of biological samples to FSL neurological Biobank. For patients who gave consent, additional blood samples were collected, and blood and derivatives were stored [35].

### Biomarkers assay

Blood samples were collectedin the morning, from 7 to 9 a.m., after a 12 h fast, at admission (baseline, T0) and at follow up, after six weeks of extensive multimodal cognitive and motor rehabilitation (T1). Samples were processed according to the biobank’s standard operating procedures (SOP), after signing of the informed consent [36].

Serum concentrations of Neuron Specific Enolase and BDNF were measured using NSE Human (Neuron-specific Enolase) ELISA kit (MyBioSource) and Quantikine ELISA - Total BDNF (R&D Systems), after validation tests to assess reproducibility and accuracy by serial dilution. All samples were diluted 1:100 and analysed in duplicate. Colorimetric reactions were measured by reading the absorbance at 450 nm after the addition of a stop solution using Varioskan LUX Multimode Microplate Reader (Thermo Fisher).

Levels of Aβ_42_, Aβ_40_, tau and NfL were assessed by a single-molecule array (SiMoA.

SR-X analyzer; Quanterix Corp., Billerica, MA, United States) using a SiMoA Human Neurology 3-Plex Advantage kit A assay (N3PA, REF 101995) and SiMoA NfL Assay, as previously reported (37)].

### Statistical analysis

A repeated measure ANOVA was used to assess changes in scales between the 2 datapoints (T0 and T1). Univariate linear regression was performed to predict changes in clinical scales between T1 and T0 (computed as difference between T1 and T0) according to baseline biomarkers levels. For analysis and data visualization purposes Matlab (version R2023a), JASP (version 0.19.2) and Graphpad were used.

**Table 1:** Demographics, clinical, and biological data of the stroke study population

Demographic data			
Total population, n	20 (9 female)		
Age	65.57 ± 16.81 years		
Days elapsed between event and hospital admission	13.56 ± 7.55		
Body Mass Index (BMI)	25.7 ± 3.8 kg/m²		
Clinical Scale	**T0**	**T1**	**p-value**
FAC	0.37 ± 1.03 (0–4)	1.68 ± 1.60 (0–5)	*p* < 0.001 (*)
RMI	1.56 ± 1.99 (0–8)	5.86 ± 4.56 (0–14)	*p* < 0.001 (*)
FMA TOTAL	77.69 ± 29.02 (36–126)	83.73 ± 31.92 (31–126)	*p* < 0.001 (*)
FMA upper limb	27.46 ± 22.37(4–66)	33.68 ± 23.73 (4–66)	*p* = 0.002 (*)
Barthel Index	18.87 ± 15.42 (2–67)	48.47 ± 27.02 (7–90)	*p* < 0.001 (*)
Biological markers			
NSE	74.92 ± 67.12	46.57 ± 54.93	NS
BDNF	39.32 ± 17.77	34.26 ± 15.11	NS
Aβ_40_	81.81 ± 58.50	75.39 ± 37.67	NS
Aβ_42_	3.01 ± 1.94	2.86 ± 1.46	NS
Aβ_42_/Aβ_40_	0.05 ± 0.03	0.038 ± 0.003	NS
NfL	1081.86 ± 870.21	N.A.	
Tau protein (total)	3.74 ± 2.77	1.89 ± 0.62	NS

*FAC* Functional Ambulation Categories, *RMI* Rivermead Mobility Index, *FMA* Fugl-Meyer

Assessment-full scores; FMA: Fugl-Meyer Assessment; NSE: Neuron Specific Enolase; BDNF: Brain Derived

Neurotrophic Factor; n, number; Age is expressed in years as Mean ± SD; Body Mass Index (BMI) is expressed as mean Mean ± SD; Clinical scales are expressed as Mean ± SD (Min - Max); biomarkers are expressed as Mean ± SD; T1, 6/8 weeks after enrollment (T0); NSE and BDNF are expressed in ng/mL; Aβ_40_, Aβ_42_, Aβ_42_/Aβ_40_, NfL and Tau protein are expressed in pg/mL

NA: not available

*Significant differences; NS: non significative differences

### Ethical Approval

 The study was conducted in agreement with ethical principles of Helsinki declaration, after the approval of the local committee (protocol number and CE/PROG.796 04-12-19).

## Result

### Patient demographics

**Fig. 1:**
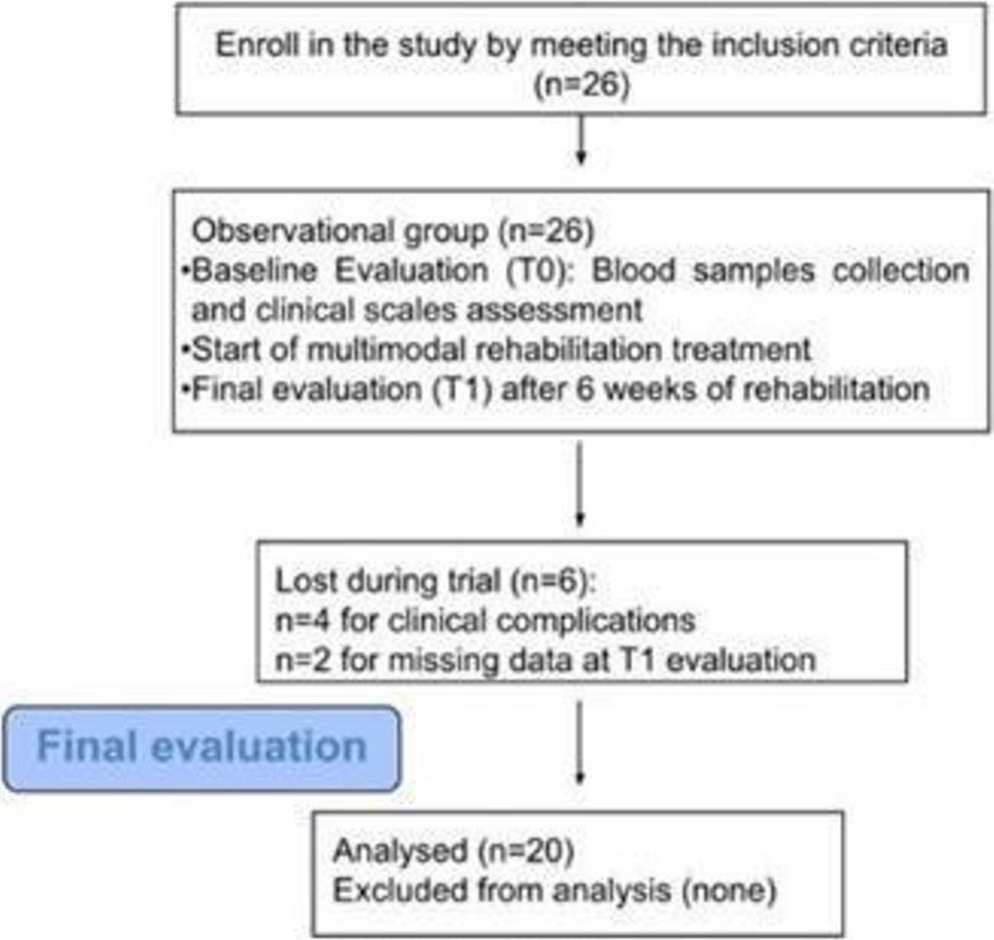
Flowchart detailing the patient selection process for post-stroke rehabilitation study

 Twenty-six patients with stroke met the inclusion criteria and were enrolled in the study, as shown in Fig. 1. Four patients dropped out due to clinical complications: one due to intestinal obstruction, two due to recurrent stroke, and one due to myocardial infarction. After the exclusion of two additional participants due to missing data, a total of 20 patients were included in the final analysis. The sample had a mean age of 65.57 ± 16.81 years, and participants were recruited between 7 and 31 days following the stroke event. Sex distribution was 11 (55%) males and 9 (45%) females. In 81% of patients, strokes were localized in the right hemisphere and the remaining 19% in the left hemisphere. 33% of events were of embolic origin while 67% originated from carotid obstruction. The clinical baseline characteristics of the subjects included in our study are given in Table1.

### Motor scales and recovery 

 A repeated measure ANOVA was used to assess changes in scales between the 2 datapoints (T0 and T1). As expected, there was a significant effect of time across all scales, representing an improvement over time: FAC, F(1, 20) = 19.310, *p < 0.001; RMI, F(1, 20) = 23.325, *p < 0.001; FM Upper limbs, F(1, 20) = 11.941, *p = 0.002, FM total; F(1, 20) = 4.979, *p = 0.037, FM total; F(1, 20) = 32.631, *p < 0.001, Barthel Index F(1, 12) = 20.817, *p < 0.001.

### Longitudinal evaluation of biomarkers levels

 To evaluate whether days after stroke or time windows of sampling among patients may affect the kinetics of changes, levels of biomarkers were measured at baseline and after six weeks (T1). In all subjects, NSE and BDNF levels did not change over time between T0 and T1: NSE, Paired t-test p = 0.22, df = 7; BDNF p = 0.15, df = 7. No differences were found also in levels of Aβ_42_, β_40_ and total tau in a subgroup of patients (n=6) (Table 1). 

### Functional walking outcome versus NSE levels

**Fig. 2:**
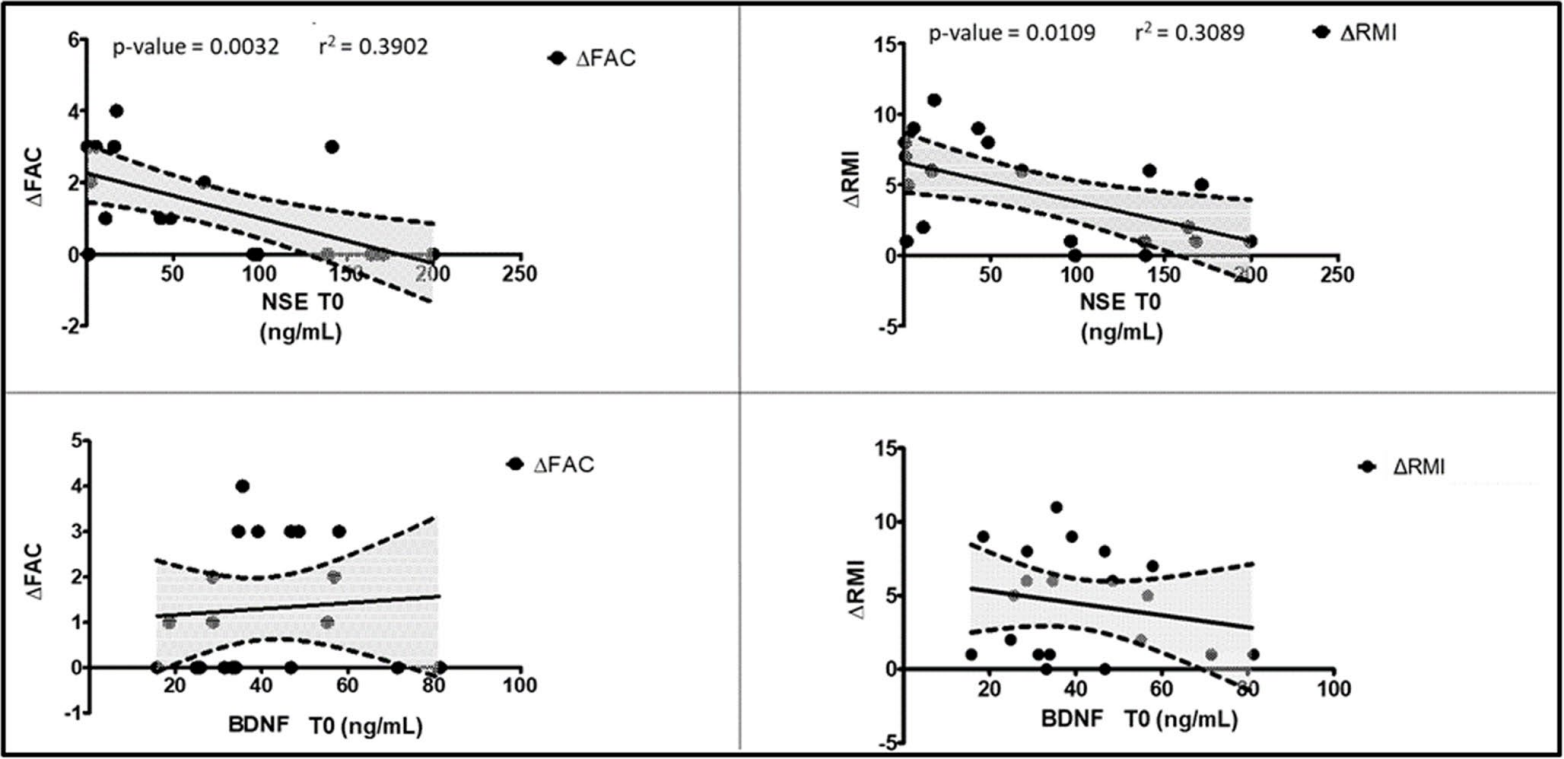
Neuronal Specific Enolase (NSE) values correlate with functional recovery in Functional Ambulation Categories (FAC) and Rivermead Mobility Index (RMI). NSE and BDNF concentrations are expressed in ng/mL; *Significant differences; NS: non significative test

 Linear regression was performed to predict changes in motor clinical scales between T1 and T0 (computed as difference between T1 and T0) according to baseline biomarker level. Among all, we found NSE at T0 was a significant predictor of improvement in FAC and RMI, where the higher NSE, the smaller the improvement (FAC: F(1, 18) = 11.5, p = 0.003, r2 = 0.390, 95% Confidence Intervals: Slope =-0.02039 to -0.004796, Y intercept = 1.462 to 3.064, X intercept=127.0 to 360.8; RMI: F(1, 20) = 23.325, *p < 0.001, r2=0.3089, 95% Confidence Intervals: Slope = -0.04836 to -0.007203, Y intercept= 4.462 to 8.690, X intercept =157.9 to 704.9). Therefore, baseline NSE explained 39% of the variation in FAC and 31% in RMI over time (Fig. 2). After including lesion side as covariate the result is as follows, and still significant (FAC: F(1, 17) = 5.5, p = 0.015, r2 = 0.392; RMI: F(1, 20) = 23.325, *p < 0.001, r2=0.3089). No significant correlation was found between the number of days passed since the acute stroke event and serum levels of NSE. Both Pearson’s correlation (r = 0.105, p = 0.649) and Spearman’s rank correlation (ρ= 0.004, p = 0.987) indicated no meaningful linear or monotonic relationship between the two variables. No association was found between recovery, as measured in FAC and RMI, and baseline levels of other biomarkers, Aβ_42_, Aβ_42_/ β_40_ ratio, total tau, NfL and BDNF (Table 1).

### Functional autonomy and daily activities outcome versus NSE and biomarkers levels

 We analyzed the correlation between biomarkers and the recovery of functional autonomy in daily activities, as assessed by the BI and FMA clinical scales, which specifically measure disability in activities of daily living and the severity of impairments in motor function involving the upper paretic limb. Linear regression analysis showed no association between baseline NSE or other biomarkers (Aβ_42_, Aβ_42_/ β_40_ ratio, total tau, NfL and BDNF) and functional autonomy recovery during the six-weeks rehabilitation period.

##  Discussion

 This study aimed to assess the ability of blood biomarkers of neuronal damage to predict improvement in functional abilities after multimodal rehabilitation, in post-acute stroke patients. Serum levels of NSE, BDNF, and classical biomarkers of neurodegeneration, Aβ_42_, Aβ_42_/ β_40_ ratio, total tau and NfL, were measured in patients at baseline, that is when the patient had been stabilized and were transferred to the post-acute stroke neurorehabilitation unit, before starting 6 weeks of intensive rehabilitation program. We found that low levels of NSE, but not other markers, were a significant predictor of good neurological outcomes, particularly for recovery of general mobility and walking, but not of daily activities and upper limb mobility, which often remains more compromised after a stroke.

 Here, we used both manual enzyme-linked immunoassays, named ELISA, as well as ultrasensitive semiautomated SiMoA, for the measurement in blood samples from patients after stroke. The observed concentrations are consistent with data from literature; in particular, NSE levels we measured (74.92 ± 67.12 ng/ml) are in accordance with other previous reports in acute ischemic stroke and other cerebrovascular diseases patients, which found correlation with early functional outcome (NSE mean levels: 64.39 ± 49.71 ng/ml) [38]. Notably, in two separate studies, NSE levels higher than 30 or 40 ng/ml, comparable with those that we measured, are directly indicative of infarction-related brain damage [39, 40] .

 Results on BDNF levels in stroke patients are vary, mainly being affected by the time since stroke event (within 10 days or longer), matrix (plasma or serum) or method used. Levels in our cohort (ranging from 26.68 and 57.88 ng/ml) were substantially similar to those reported in other studies in plasma or serum in acute stroke (6.69 ± 4.92 ng/ml and 9.93 ± 4.04 ng/ml) [41, 42]. Comparable BDNF levels were measured in a study on 660 ischemic stroke patients, with BDNF higher than 38.61 ng/ml inversely associated with severity of post stroke cognitive impairment [43]. 

 Similarly, levels of the other biomarkers, assessed using SiMoA technologies, were comparable with other studies on stroke and other neurological patients. NfL levels (1081.86 ± 870.21 pg/ml) were in agreement with values typically observed within a three month post-stroke period [44] (Median = 73.45 pg/ml, interquartile range (IQR) 93.95 pg/ml]. Likewise, our reported blood-derived tau protein levels at T1(3.74 ± 2.77 pg/ml) were consistent with previous findings (Median, 5.0 pg/ml and IQR, (2.6 - 10.3) ; Median 16.0 and IQR (14.4 - 17.9)) [45, 46]. Finally, our Aβ_40_ and Aβ_42_ concentrations (Aβ_40_= 81.81 ± 58.50 pg/ml, Aβ_42_= 3.01 ± 1.94 pg/ml) were in accordance with similar studies (Aβ_40_: 90.1 ± 70.9 pg/mL, Aβ_42_: 2.3 ± 3.2 pg/mL; Aβ_40_>7 days: Median 16.0 and IQR (14.4 - 17.9) pg/ml) [37, 46].

 These findings suggest that specific neurological markers may be assessed in clinical setting by using validated assays, and be used as surrogate index of neuropathological processes, with important clinical implication. In our study, evaluating NSE in a neurorehabilitation setting after stroke can predict significant motor improvement in the medium-short term, as assessed by FAC and RMI scales, where the higher NSE, the smaller the improvement. Moreover, we observed that baseline NSE explained 39% of the variation in FAC and 31% in RMI over time; whereas, no significant association were observed with respect to other clinical scales for functional autonomy and daily activities, or other biomarkers. Similarly, in AD patients cerebrospinal fluid Aβ_42_ levels, but not total tau and p-tau, significantly correlate with severity of extrapyramidal signs, with gait and mobility, and with static and dynamic balance (evaluated by Unified Parkinson’s Disease Rating Scale (UPDRS) part 3; Rating Scale for Gait Evaluation in Cognitive Deterioration (RSGECD); and by Tinetti scale score, respectively) [47]. In de novo Parkinson’disease patients, elevated CSF ptau/tau is a predictor of higher risk of development of motor complication [48]. This suggests the importance of biomarker-based stratification, and use of circulating brain markers of proteinopathies, amyloid metabolism, to investigate motor characteristics caused by neuronal damage. 

 Several factors affect stroke risk and rate of recovery, such as biological and genetic variability, individual factors, comorbidities, as well as the severity of the lesion, etc. [49, 50]. Recently, clinical studies and meta-analyses have highlighted correlations between biomarkers level, the extent of poststroke damage and neurological outcome, including death, supporting the potential as a prognostic biomarker. A systematic review of eleven studies, on a total of 1398 ischemic stroke patients [18], found a significant correlation between NSE levels and severity of neurological symptoms, deficits and mortality rates [49, 51, 52], with exception of only one study [31]. Notwithstanding, data on NSE and functional outcomes gave conflicting results, with some studies reporting statistical significance between blood levels and recovery at discharge or in the early days after stroke, assessed by the modified Rankin Scale (mRS) [51, 53] a commonly used scale to measure the degree of disability or dependence in daily activities after a stroke event, whereas others founding no association [54–56]. Accordingly, we found that NSE levels do not correlate with the Barthel index and FMA, which include items related to functional autonomy, such as dressing and eating. In such functional activities of daily living, the involvement of the upper limb is necessary, which instead shows a poor recovery in stroke patients, as demonstrated by FMA in our group of patients. Instead, the FAC and RMI items include the prevalent involvement of the trunk and lower limb, with FAC specifically evaluating walking and ambulation ability, and the human support the patient requires when walking, regardless of whether or not they use a personal assistive device [31]; and RMI scale quantifying mobility disability, testing functional abilities such as gait, balance, and transfer [57]. We can therefore hypothesize that NSE levels may be an indicator of favorable recovery, but limited to simpler functions, such as walking; while they do not correlate with the recovery of more complex daily activities, which require, for example, the use of the upper limb. In this context, biological markers, such as NSE, could be valuable in an integrated approach for stroke patients, by combining neuroimaging, neurophysiology and clinical evaluation, to predict neurological and motor outcomes, as well as response to therapies. This is crucial to define personalized multimodal rehabilitation plans, including predicting proportional recovery from motor deficits across various patient categories. Importantly, proportional recovery may reflect the biological mechanisms underlying repair of damage, which may be assessed by the measure of specific biological markers.

 Unfortunately, we found the levels of other biomarkers, including BDNF, Aβ_42_, Aβ_42_/ Aβ_40_ ratio, t-tau and NfL, showed no association with neurological or motor recovery as measured in the used clinical scales. This may be partly due to different characteristics of the markers analyzed, which reflect different features of the complex mechanisms of neuronal damage and repair, as well as to population heterogeneity. However, the literature data on BDNF and stroke are varied and discordant [43, 58]. One reason for the lack of association with motor recovery could indicate a stronger link between BDNF, neuroplasticity [59] and cognitive protection from stroke-related damage, as opposed to motor and functional recovery, since BDNF levels higher than 38.61 ng/ml were inversely associated with post stroke cognitive impairment severity [43]. On the other hand, the lack of association with amyloid may be attributed to mechanisms different than amyloidopathies occurring in cerebrovascular pathologies, whereas the same absence of correlation of tau and NfL with stroke-related brain damage warrants further investigation.

 This study has major limitations that are the absence of standardized stroke severity measures (e.g., NIHSS, infarct volume) and the small sample size and which limit the generalizability of this work. Although neuroimaging studies suggest a correlation between NSE levels and the extent of stroke lesions, unfortunately, we were unable to assess association between functional recovery and NSE levels in relation to initial NIHSS values, which could have provided further valuable insights into this topic. Therefore, this work should be considered an exploratory study on the role of NSE in stroke recovery.

 Regarding the sparse number of patients, we recruited subjects characterized by a high disability at hospitalization (average Barthel Index 18.87) with ischemic stroke that reached clinical stability between 7 up to 30 days from stroke event. Although this heterogeneity over time may be considered a limitation, it nevertheless takes into account an important clinical aspect, namely individual variability needed for stabilization following stroke and, presumably, also the associated biochemical and inflammatory processes.

 Since studies from literature to date have shown conflicting results regarding biomarkers correlation, including NSE, with various stroke scale scores, infarct volume and outcome parameters, several issues need to be considered. One reason may be that patient selection and time since stroke represent a huge source of heterogeneity, thus affecting reproducibility and comparability in multicenter studies. Indeed, NSE release after stroke has a specific window, which increases 2-3 hours after the onset of the first stroke, then decreases, followed by a secondary increase until day 5, probably reflecting a secondary mechanism of brain damage and ongoing neuronal cell death [60, 61]. Of note, we found no significant correlation between the number of days passed since the acute stroke event and serum levels of NSE, nor differences at baseline and at six-weeks follow up. These findings suggest that NSE serum concentrations do not show a consistent trend over time following stroke in the dataset analyzed. In addition, the use of various techniques, e.g. commercial ELISA tests, with different detection thresholds, sensitivities and specificities represent a source of variability among measurements of serum NSE concentrations among studies. From a clinical perspective, different stroke scales are used, which may differ in items and domains or specific skills measured. For example, we found a specific correlation with gait recovery, measured by FAC and MRI, but not with autonomy and recovery, assessed by Barthel Index and Fugl Mayer. Longitudinal studies on large cohorts are needed to evaluate the usefulness of serum NSE in the clinical context, to predict recovery in motor functions or even in other cognitive domains, such as attention and communication, in combination with other biochemical or morphological markers. In future studies, neuroimaging data and systematically collect NIHSS/mRS scores should be integrated to strengthen the clinical relevance and predictive value of blood biomarkers in post-stroke recovery.

 In conclusion, low blood NSE in subacute ischemic stroke patients suggests a potential good outcome, with high proportional motor recovery, while there is no association with BDNF, Aβ_42_, Aβ_42_/ Aβ_40_ ratio, t-tau and NfL. Research on novel blood biomarkers seems promising to improve clinical care and rehabilitative strategies.

## Data Availability

The datasets analyzed during the current study are available from the corresponding author on request.
